# I Will Hurt You for This, When and How Subordinates Take Revenge From Abusive Supervisors: A Perspective of Displaced Revenge

**DOI:** 10.3389/fpsyg.2020.503153

**Published:** 2020-09-23

**Authors:** Li Hongbo, Muhammad Waqas, Hussain Tariq, Atuahene Antwiwaa Nana Abena, Opoku Charles Akwasi, Sheikh Farhan Ashraf

**Affiliations:** ^1^School of Management, Jiangsu University, Zhenjiang, China; ^2^NUST Business School, National University of Sciences and Technology, Islamabad, Pakistan; ^3^Management Science and Engineering, School of Management, Jiangsu University, Zhenjiang, China

**Keywords:** abusive supervision, social exchange theory, displaced revenge, service sabotage, perceived supervisors’ remorse

## Abstract

Abusive supervision, defined as subordinates’ perception of the extent to which supervisors engage in the sustained display of hostile verbal and non-verbal behaviors, excluding physical contact, is associated with various negative outcomes. This has made it easy for researchers to overlook the possibility that some supervisors regret their bad behavior and express remorse for their actions. Hence, we know little about how subordinates react to the perception that their supervisor is remorseful and how this perception affects the outcomes of supervisors’ undesired behavior. Specifically, drawing on the social exchange theory (SET) and displace revenge literature, this study explains how abusive supervision leads to victims’ service sabotage behavior. In addition, this study also investigates how perceived supervisors’ remorse (PSR) mitigates the adverse effects of abusive supervision. Based on time-lagged, dyadic data (63 supervisors, 212 subordinates) from Chinese individuals, this study found support for all the proposed relationships, i.e., abusive supervision leads to service sabotage through the mediating effect of revenge desire. The findings also conclude that PSR lessens the detrimental effects of abusive supervision on victims’ behavior with their customers. Finally, this research contributes to service sabotage literature by highlighting the possibility where abusive supervisors cause service sabotage behavior among victims. This study also shows the importance of PSR’s role in decreasing service sabotage behavior exhibited by victims of abusive supervisors in the service sector.

## Introduction

Employees’ ethical behavior is one of the key factors for an organization to be effective and efficient. This factor becomes more crucial for in-service sector organizations as they continuously interact with their customers and their effectivity depends on their customers’ perceived satisfaction. That is, these are the customers who perceive whether interactions are highly ethical and where they can bring in new customers through positive word-of-mouth referrals. On the other hand, customers’ perceptions of inappropriate dealing can also hurt the service organizations’ chances to attract new customers. Unlike satisfied customers, dissatisfied customers quickly disseminate information about the negative experience they had. Reputation is then tarnished, which affects the financial standing of the organization. Yet as employee’s behavior is critical to organizational effectiveness, service sabotage has been described as employees’ widespread deliberate misbehavior. In a quest to highlight widespread sabotage behavior, Harris and Ogbonna (2002) reported that 85% of employees are involved in sabotage behavior. They further stated that service sabotage has the potential to exert long-term, detrimental effects on the reputational and financial capital of the organizations. Moreover, it also dents the organizational image that leads to a reduction in service patronage, thereby affecting the financial status of such organizations. As mentioned, service sabotage costs US firms up to $200 bn per year (Hongbo et al., 2019).

These far-reaching, damaging effects of service sabotage have generated an interest to identify its antecedents. Besides, keeping in mind the widespread incidents and the decline of billions of dollars every year, it is important to highlight such factors that encourage employees’ involvement in sabotage behavior. Customer negative event has been highlighted as a major antecedent of employees’ sabotage behavior (Hongbo et al., 2019). However, this study takes a new perspective by considering supervisors’ mistreatment as an antecedent of service sabotage. This study takes the focus off customers and highlights the impact of supervisors’ behavior on employees’ interaction with their customers. Though several studies (Cortina and Magley, 2009; Crossley, 2009; Martinko et al., 2013) examined the workplace mistreatment and the fact that interpersonal relations in the workplace consist of the important factor in regulating employees’ behavior, there exists a gap to address how supervisors’ mistreatment affects employees’ interaction with customers. This paper, therefore, seeks to revise, update, and enlarge the literature in this area.

As mentioned, the focus of this study is on supervisors’ mistreatment. Specifically, this study is based on supervisors’ undesired behavior, i.e., abusive supervision, defined as “subordinates’ perception of the extent to which supervisors engage in the sustained display of hostile verbal and non-verbal behaviors, excluding physical contact” (Tepper, 2000, p. 178). Such abusive behavior is frustrating, which then makes emotions set in, and in all, it affects subordinates’ behavior. The fact that a supervisor possesses power does not mean power obsession should drop in the dealings with subordinates. Thus, abusive supervisors violate the purpose of power, and authority conferred on a supervisor. The research has documented the negative effects of abusive supervision on both individual and organizational outcomes. For instance, abused subordinates become emotionally exhausted (Wu and Hu, 2009) and exhibit deviant behavior (Tepper et al., 2008). It is also a costly workplace phenomenon as well: 14% of workers are estimated to have experienced abusive supervision, resulting in an estimated cost of $24 billion annually to organizations (Xu et al., 2012). With these far-reaching consequences, this study considers abusive supervision as one of the possible antecedents of employees’ service sabotage behavior.

Furthermore, empirical research (Thau et al., 2009; Martinko et al., 2013) on abusive supervision has focused on aggressive responses by subordinates. However, it is important to note that employees do not always respond with the same intensity. If that is the case, there arises a logical question: Under which circumstances are abusive supervision effects mitigated? One possible reason could be the supervisors’ remorse, defined as feelings of regret in an attempt to repair the loss that has occurred because of some undesired behavior by supervisors (Weisman, 2016). In light of this, we consider the supervisors’ perceived remorse and propose that it curbs the intensity of subordinates’ response to mistreatment by their supervisors. Specifically, we theorize that those employees who perceive that their supervisors are repentant for their abusive behavior and are willing to rebalance the exchange relationship with their subordinates experience less revenge desire, which limits their service sabotage behavior.

This study contributes to the literature in several ways. First, it highlights the possibility that supervisors who exhibit abusive behavior may also feel remorse (PSR) for their abuse; this is a possibility (along with its attendant implications) that seems to have been neglected so far. Second, by integrating social exchange theory and displaced revenge literature, this study identifies abusive supervision as a precursor of employees’ service sabotage behavior. The past research has primarily highlighted the customers’ negative events as a major antecedent of service sabotage. Lastly, by examining an organizational outcome (i.e., service sabotage), we extend research on revenge desire as a mechanism through which abusive supervision influences subordinates’ behavior with their customers. While this is not the only mechanism that can serve as an explanation for this phenomenon, this study proposes that victims’ revenge desire mediates the positive relationship between abusive supervision and service sabotage. Hence, this study not only highlights the abusive supervision as an antecedent of service sabotage, the mechanism through which it occurs, and the moderating effects of PSR but also provides insights to organizations for managing service sector employees so they can effectively interact with customers even under abusive supervision. Organizations with such insights will be much more prepared to handle service sabotage behavior.

**FIGURE 1:**
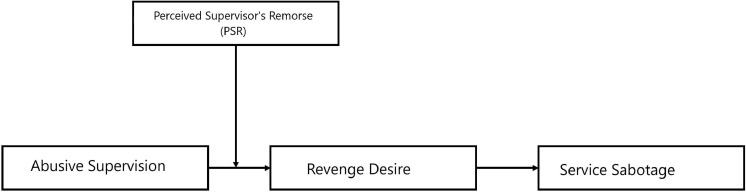
The proposed moderated mediation model.

Looking upon the intuition from SET and displaced revenge literature, the hypotheses are developed first, i.e., a positive association among abusive supervision and service sabotage through mediating effect of revenge desire that describes “how abusive supervision guides toward the service sabotage”? Next, we speculate the slackening role of perceived supervisor remorse to answer “how employees’ perception of their supervisors’ remorse could moderate the positive association between abusive supervision and revenge desire”? The broader contributions to theory, practical implications, and suggestions for future research are discussed. Our proposed moderated mediation model is depicted in Figure 1.

### Literature Review and Hypothesis Development

Social exchange theory (SET) is considered one of the oldest theories of social behavior and claims that any interaction between individuals is an exchange of resources. These exchanged resources may not only include tangible items such as goods or money but could also include intangibles such as social relations including friends and relatives. SET (Homans, 1958; Blau, 1964) suggests that social exchanges are “voluntary actions” that may be initiated by one’s treatment with others, with the expectation that such treatment will eventually be reciprocated (Davies and Gould-Williams, 2005). The exact nature and extent of future returns are dependent on the discretion of the person making them and are thought to be a function of personal obligation, gratitude, and trust in others. The theory further suggests that social exchanges are derived from informal relationships that create personal feelings of trust and obligation. A social exchange relation also refers to an enduring interaction pattern rooted in mutual obligations and commitment to the other party’s needs. Eisenberger et al. (1990) explained how the process of social exchanges is initiated by organizations when a general perception concerning the extent to which the organization values general contributions and cares for their well-being is achieved. The basic assumption of the theory, which states that parties enter into and maintain relationships with the expectation that doing so will be rewarding, is also applicable to the organization-employee relationship where employees perceive that organizations value and deal equitably with them, and they will reciprocate these “good deeds” with positive work behavior. Gouldner (1960) also supported the fact that social exchanges are dependent on actors orienting themselves toward a general norm of reciprocity. For instance, people return favors by engaging in cooperative behavior (i.e., positive reciprocity). The same law of reciprocity also applies to such acts in which individuals return what they perceive to be a negative treatment by retaliating. In this way, SET attempts to explain individuals’ behavior with each other by claiming that it depends on the nature of exchanges between them. Since the development of SET, research has empirically supported its basic tenets. For example, numerous studies (Mitchell et al., 2012; Shiau and Luo, 2012; Ko and Hur, 2014) have been conducted to explain how the nature of exchanges affect individuals’ behavior.

This research leads to the abusive supervision literature and standards that the abusive behavior of the supervisor is associated with the social interchange among supervisors and their employees. The supervisors within any organization have great influence over the work lives of subordinates by shaping their (subordinate) experience through the methods they (supervisor) utilize to allocate resources, assign tasks, and how they manage their interpersonal interactions (Hutchinson, 2015). The behavior and attitude of the supervisors emphasize a lot on the social exchange relationships that the employees build with the supervisors, i.e., when supervisors are helpful and polite, the employees will be better able to respond properly. While the abusive supervision is known as “‘subordinates’ thought of the limit to which supervisors get involved in the aggressive verbal and non-verbal behaviors, without causing any physical harm” (Tepper, 2000, p. 178) is contrary to that which breaches the laws of social exchanges. For example, whenever a supervisor practices abusive supervision by talking rudely to the employee or by insulting them, it hurts the expectations of the other party, which may result in undesired behavior. It is a prevalent form of mistreatment that has received growing interest in research (Schyns and Schilling, 2013; Zhang and Bednall, 2016), which reported that abusive supervisors are toxic for subordinates and also cause negative externalities for the organization in the form of behavioral consequences, one of which is increased employees’ turnover, increased strain, decrease in effective welfare, and low-quality social exchanges (Lian et al., 2012; Wheeler et al., 2013; Tillman et al., 2018). It is also positively associated with subordinates’ tendencies to engage in dysfunctional manners at work (e.g., workplace deviance; Mitchell and Ambrose, 2007). Furthermore, subordinates who perceive abuse from supervisors establish lower levels of work performance and organizational citizenship behavior than their counterparts (Zellars et al., 2002; Xu et al., 2012).

After experiencing abusive supervision, individuals undergo an appraisal process where the social exchange is evaluated relative to expectations. For them, inappropriate treatment breaks the norm of mutual respect and, hence, evokes feelings of injustice. Furthermore, it also signals that the supervisor does not value and accept them, which threatens subordinates’ social standings. Such perceptions lead to victims’ reactions through negative intentions. Here, we consider one such intention, i.e., revenge desire. Revenge desire, either deliberate or not deliberate, occurs because of inflicted interactions in which the victim thinks there should be justice and equity. In short, it occurs as payback for an already happened negative incident. The motivation is revenge, not because human beings are fundamentally evil, but because vengeance is part of the innate survival mechanics of a complex social species. Reciprocity or “tit-for-tat” is the basis of social relationships, manifesting even among our primate ancestors, i.e., a tit for tat case. Summarizing the debate, in line with SET, we propose that abusive supervision is against the norms of social interactions, which elicits negative desires (revenge desire).

When an employee is filled with revenge desire, he or she plans for equity and justice. The sufferers may rationalize their revenge by declaring they are already done with their fair share of trouble—as they were the only part of the victimhood who have been suffering from ages (Zitek et al., 2010). But, when there is an imbalance of power between the perpetrator and the victim, then the bearer of injustice does not reciprocate the negative behavior to the original perpetrator and rather diverts his/her revenge toward innocent targets. For that, we theorize that the subordinates do not respond to their supervisors because the complex power dynamics that exist in an organization make it difficult for employees to respond to a supervisor’s maltreatment without fear of bringing upon themselves direct and costly punishment. If the subordinates revert toward their supervisors, there are chances of the employees being transferred, demoted, or even fired. With these fears, the employees ultimately divert their revenge away from supervisors, i.e., displaced revenge.

Displaced revenge, defined as a retributive reaction toward a prior transgression that is not directed against the original transgressor(s), but rather against uninvolved targets. While, at first glance, it seems irrational and morally questionable to take revenge from those who were not personally involved in the initial transgression, however, when victims cannot directly punish offenders, the ultimate option left for them is to divert their revenge to the next best option and lash out at people who are easy to target, in other words, displace their revenge (Bushman et al., 2005; Denson et al., 2006). Here, we state that the victims of abusive supervision displace their revenge desire to the nearest possible entity, which could be the coworkers or the customers (in the case of the service industry). Nevertheless, the employees usually do not express their dissatisfaction to coworkers because on the level of power and functioning, the coworkers are seen at the same level as the revenge-taking employee, and if there is a transfer of any dissatisfaction to the coworkers, the coworkers can bounce back that same action to the avenging employee, which add further toxicity to the workplace environment. The ultimate option left for abused employees to displace their revenge is the customers because they are perceived as less powerful entities to receive the revenge, even though innocent. The study says that the service sector employees’ attitude mostly diverges with what is fancied (Harris and Ogbonna, 2009, 2012). That is, employees habitually put up attitudes that retrogress service quality, i.e., service sabotage. *Service sabotage*, the process of showing attitude knowingly, which happens when the employees distort the customers’ service (Harris and Ogbonna, 2006; Skarlicki et al., 2008; Chi et al., 2013), is surprisingly regular in the service section (Kao et al., 2014) and can take place regularly (Harris and Ogbonna, 2006). For instance, Harris and Ogbonna (2002) explored the fact that 85% of employees subjected to intentionally sabotaging customer services for seven days. The situations linked with such sabotage involve a decrease in customer contentment and recognized service quality, lessened customer allegiance, and weak customer devotion to the firm (Harris and Ogbonna, 2002, 2009). Additionally, it also causes a financial loss of $200 billion per year (Harris and Ogbonna, 2012; Lee and Ok, 2014). Such unfavorable circumstances show the significance of recognizing the elements that encourage employees to get involved in these types of intentional impractical attitude. In a search to highlight one possible factor, this study positions that in the matter of abusive supervision, the exploited sufferers feel abused but are unable to fight back against the actual provocateur because of the power imbalance between them. Making the fact simpler, the prediction of a highly traumatic surrounding hinders the acts of direct revenge and enables the practice of displaced revenge. Thus, it is hypothesized that the abused employees shift their revenge will away from their supervisors and implement it on the customers who are an easy and guilt-free target available in the service industry. By doing so, this research is the first one to merge social exchange theory and displace revenge literature to explain how the flawed social exchange between supervisor and subordinates can impact the customers to a larger extent. We suggest that the abusive supervision initiates revenge desires in victims, rather than challenging the actual source of victimization, and employees then turn their revenge toward customers and sabotage their services.

***H1*.** Revenge desire mediates the positive relationship between abusive supervision and service sabotage.

### Moderating Effect of Perceived Supervisor Remorse

Remorse and apology are two different terminologies that have been used interchangeably in a different contest of our daily lives. Verbal expression of remorse is a pivotal aspect of apology while an apology is seen as important but not a condition that suffices remorse (Weisman, 2016). An individual’s or individuals’ sincere acceptance and acknowledgment of the unfair act is termed as an apology. Remorse, on the other hand, is the behavioral expression that the offender demonstrates by expressing empathy and being sorry for engaging in hurtful conduct toward others and is accompanied by verbal expression. Weisman (2016, p. 10) described, “while an apology may refer to the anguish and pain that the offender feels at having contravened the norms of the community, an expression of remorse shows or demonstrates this pain by making the suffering visible.” In other words, an apology is expressed verbally, whereas remorse is expressed behaviorally as well as verbally, such as “gestures, display of effect, and other paralinguistic devices” (Weisman, 2016, p. 10). Weisman (2016) further identified three necessary conditions for remorse that include willingness to accept one’s hurtful behavior and the damage it caused, expressing empathy of visible suffering for one’s hurtful behavior, and the intention to make corrections to avert future occurrence of such hurtful behavior. We can conclude from the above explanation that remorse not only focuses on verbal expression but also considers the actions and gestures that depict genuine intentions and feelings of the offender to repatriate for the harm done. This study accurately assimilates the discerned supervisors’ guilt, which is the subordinates’ idea about their supervisors’ repentance.

Perceived supervisors’ remorse (PSR) is defined as “a subordinate’s perception that their supervisor is experiencing feelings of personal guilt and regret about supervisor’s behavior toward the subordinate” (Haggard and Park, 2018, p. 2). This definition of supervisors’ remorse integrates supervisors’ acceptance of their hurtful behavior and their desire to avoid such hurtful behavior in the future. Several studies (Davis and Gold, 2011; Haggard and Park, 2018) have provided evidence-based reasons for remorse to be effective in repairing the damage caused by wrongful behavior. For instance, attributional perspective argues that regretful and sincere remorse reduces the victim’s internal attributions for wrongdoer’s behavior and strengthens his/her belief that such behavior is not likely to happen again. According to some classics (Tomlinson, 2012), the wrongdoer could express remorse to the victims that would make the victims generate some empathy resulting in forgiving the wrongdoer to repair their relationship. Concerning this situation, the victim knows the importance of their relationship and the need to value it by maintaining it through reparation. Justice literature has also proven the effectiveness of remorse in repairing a relationship, for instance, interactional justice refers to the expectation one has that he/she would be respected, treated fairly, and honored by organizational agents. Abusive supervision, on the other hand, is the violation of interactional justice by supervisors that affects the exchange relationship between supervisors and their subordinates (Barnes et al., 2015), and is taken as disrespectful and unfair. Nevertheless, remorse creates a perception of interactional justice and repairs shaken trust (Walfisch et al., 2013). Subordinates attribute supervisors’ abusive behavior as impulsive rather than intentional when they perceive the supervisor’s remorse. According to Walfisch et al. (2013), managers who show remorse are more effective in repairing workplace offense. This is because subordinates least expect remorse from supervisors; thus, the least expected they were, the more their effectiveness.

Based on the above arguments, we assume that the expression of remorse at supervisors’ part reduces the negative effects of supervisors’ abusive behavior. This is because the supervisor’s remorse indicates they accept responsibility for their actions and are prepared to repair the damaged relationships with their subordinates. It also represents the supervisors’ willingness to prevent the future occurrence of their hurtful behavior, which mitigates the effect of infringing of interactional justice. That is, the remorseful apology tends to restore interactional justice. Our research proposes that the perceived supervisor’s remorse will weaken the relationship between abusive supervision and revenge desire.

***H2a***: PSR will moderate the direct positive relationship between abusive supervision and revenge desire such that the relationship will be weaker when PSR is high.
***H2b***: PSR will moderate the indirect positive relationship between abusive supervision and service sabotage through revenge desire such that the mediated relationship will be weaker when PSR is high.

## Methodology

### Participants

The respondents were employees in a cellular company operating in China; however, the data was collected from 21 offices located in Jiangsu, Hubei, and Anhui Province. These offices were selected based on convenience sampling technique. We communicated with human resource (HR) officials of the company to support this study and to encourage employees’ participation. To minimize the potential effects of the common bias method (Podsakoff et al., 2003), data were collected at three different times each with a 1-month gap. For that, three different surveys were designed: Survey-1 was designed for employees to provide demographic and control variables information, supervisors’ abusive behavior, and perceived supervisors’ remorse. Survey-2 was designed to collect information about employees’ revenge desire, whereas survey-3 was designed for supervisors to rate their subordinates’ sabotage behavior. Overall, 101 supervisors and 389 subordinates participated in this study. However, we collected a total of 72 supervisors administered surveys with a response rate of 71% and 269 subordinates administered surveys with a 69% response rate. Following the prior research (e.g., see Tariq et al., 2019) on supervisor-subordinate dyadic data, only those groups were considered in which at least two subordinated rated their respective supervisors. Finally, a total of 63 supervisors and 212 subordinates with average group size 3.35 participated in this study.

Among final participants, 62% were female, the mean age was 28.6 years, and their average work experience in the service industry was 2.8 years. Most (56.7%) of the participants were university graduates.

### Measures

The parameters used in this research were authentically created in English. Following the practice of “double-blinded principle” (Brislin, 1980), we used conventional “translate-back translate” method to convert the English language survey into the Chinese language, and this method was applied to reinforce the reliability and validity of the measures (Yang et al., 2000). We requested two Chinese bilingual professors to do the “translate-back-translate” process independently, and then 12 subordinates of three supervisors (not part of our sample) were requested for the pretest and constructive feedback for Chinese survey modification (Aryee and Chen, 2006).

### Abusive Supervision

Abusive supervision was measured using Tepper (2000) 15−item scale, which asks respondents to indicate how often their supervisors used certain behaviors (1 = never, 5 = always). Sample items include, “My supervisor tells me my thoughts or feelings are stupid,” and “Is rude to me.” Alpha reliability was 0.88.

### Employee Service Sabotage

Many service sabotage measures were created to use in a call center setting. While, there is big dissimilarity in the services offered by call center agents, and those who deal face-to-face with the customers. Along with such doubts in mind, we calculated service sabotage using the Chi et al. (2013) three-item scale. Statements such as “mistreating customers deliberately” were measured on a five-point attribution scale (1 = never, and 5 = always). The Cronbach’s α for this scale was 0.77.

### Perceived Supervisor Remorse

Perception of supervisor remorse was measured using Haggard and Park (2018) 10-item scale, which asks respondents to indicate how often their supervisors used certain behaviors after they (supervisors) had done something hurtful: “Admitted that his/her behavior was unacceptable,” “Took responsibility for his/her hurtful behavior,” “Asked what he/she could do to repair the damage to your relationship,” and “Expressed that he/she felt bad about how his/her behavior affected you” (1 = never, and 5 = always). Alpha reliability was 0.74.

### Revenge Desire

Revenge desire was measured using Jones (2009) four-item scale. Responses were recorded on a five-point Likert scale (1 = strongly disagree and 5 = strongly agree). Among four, two items were intended to assess retaliatory intentions, i.e., “I intend to settle the score with my supervisor,” and “I plan on getting even with my supervisor soon.” Whereas, remaining two items were intended to assess the expected utility of revenge, i.e., “If I were mistreated by my supervisor the satisfaction of getting even would outweigh the risks of getting caught,” and “If I were mistreated by my supervisor it would feel good to get back in some way.”

### Control Variable

Customer negative events are considered as the most common reason for employees’ service sabotage behavior; though discouraged, it is rational to get back at those customers who act unpleasantly with company representatives. To evade its influence in our study, we controlled for customer negative events that were measured by a three-item scale adopted from Chi et al. (2013); responses were recorded on five-point Likert scale (1 = strongly disagree and 5 = strongly agree). Besides, as suggested by others (Hongbo et al., 2019), demographic factors have the potential to influence hypothesized relationships, and to avoid such misspecification, we controlled employees’ age, gender, education, and tenure in the service industry.

## Results

### Descriptive Statistics

Table 1 provides our study’s descriptive statistics (standard deviations, means, and estimated coefficient alpha values) and intercorrelations. The preliminary analyses support our hypotheses i.e., abusive supervision is positively related to revenge desire (*r* = 0.54, *p* < 0.01) and service sabotage (*r* = 0.49, *p* < 0.01). Revenge desire is also positively related to service sabotage (*r* = 0.46, *p* < 0.01).

**TABLE 1:** Intercorrelations, descriptive statistics, and estimated reliabilities among the latent variables.

**Variables**	**M**	**SD**	**1**	**2**	**3**	**4**	**5**	**6**	**7**	**8**	**9**
Subordinate gender^a^	1.32	0.46	–								
Subordinate age^b^	3.06	1.21	–0.10	–							
Subordinate education^c^	3.08	1.21	–0.03	0.11	–						
Subordinate experience in service industry^d^	2.22	1.89	−0.23**	–0.06	–0.03	–					
Customers’ negative events	2.13	1.20	–0.01	–0.04	–0.11	0.06	(0.70)				
Abusive supervision	3.43	0.64	–0.02	0.13	0.49**	–0.05	–0.09	(0.88)			
Revenge desire	3.55	0.83	–0.04	0.14*	0.28**	–0.04	−0.24**	0.54**	(0.76)		
Service sabotage	3.00	0.86	–0.04	0.08	0.20**	–0.11	0.07	0.49**	0.46**	(0.77)	
Perceived supervisor remorse	3.37	0.78	–0.02	–0.06	–0.09	0.03	–0.08	–0.01	–0.08	0.01	(0.74)

*N* = 63 supervisors and 212 subordinates (Dyadic data); Significant at: *p < 0.05; **p < 0.01; figures in parentheses are alpha internal consistency reliabilities. ^*a*^Subordinate gender: 1 = Male, 2 = Female; ^*b*^Subordinate age: 1 = Less than 24 years, 2 = 26–30 years, 3 = 31–35 years, 4 = 36–40 years, 5 = more than 40 years; ^*c*^Subordinate education: 1 = Primary education, 2 = High school, 3 = College education, 4 = Vocational education 5 = Others; ^*d*^Subordinate working experience in service industry = 1 = Less than 1 year, 2 = 1–3 years, 3 = 4–6 years, 4 = 7–9 years, more than 9 years.

### Analytical Approach

Besides the different working environments of each office, the subordinates in this study who were from the same office reported to the same supervisor. Thus, there is potential that our data may not be independent and may violate the assumption of ordinary least squares (OLS) regression, which could not result in a biased estimate of standard errors and invalid test statistics. Before testing the formal moderated mediation model, we computed the intraclass coefficient 1 (ICC1, which represents the amount of variance that resides between supervisors) and the intraclass coefficient 2 (ICC2, which represents the stability of the supervisor means) for each study variable to determine the appropriate level of analysis. The ICCs for abusive supervision, revenge desire, employee service sabotage, and perceived supervisor remorse were 0.04, 0.05, 0.19, 0.25, and 0.06, respectively. The ICC2s for the same variables were 0.11, 0.07, 0.47, 0.35, and 0.28, respectively, all of which are below the generally accepted level of 0.70. These results suggest there was an insufficient variance between supervisors coupled with low stabilities of their means to warrant using a multi-level approach. However, we also calculated a corrected (unbiased) F statistic for each of our analyses as this is a more conservative test. Results indicated that all of our corrected F statistics remained significant and decreased by no more than 0.10 (e.g., the F for leader performance ratings changed only 0.03, from 11.63 to 11.60). All of these results, coupled with Kenny’s (1995) statement that if ICC1s are below 0.3 it is relatively safe to analyze data at the individual level, lead us to the decision to analyze all variables in the study at the individual level. Therefore, we are strict with the individual level of data analysis of our study.

We first used SPSS “PROCESS macro” to test our formal mediation hypothesis (i.e., Hypothesis 1). Similar to recent previous studies (e.g., Tariq and Ding, 2018a; Tariq and Weng, 2018b; Ahmad et al., 2019; Butt et al., 2019; Hongbo et al., 2019; Shillamkwese et al., 2020; Tariq et al., 2020), and recommendations of Hayes (2013) and Preacher et al. (2007), to use model 4 of “PROCESS macro” to verify our Hypothesis 1. We then utilized model 7 of “PROCESS macro” to test our proposed moderated mediation model (i.e., Hypotheses 2a and 2b).

### Test of Mediation

Table 2 presents the findings of the mediation test. Abusive supervision is positively correlated with revenge desire (*B* = 0.68, *t* = 7.93, *p* < 0.001) and service sabotage (*B* = 0.47, *t* = 4.67, *p* < 0.001). Revenge desire is also positively correlated with service sabotage (*B* = 0.33, *t* = 4.54, *p* < 0.001). Table 2 also indicates the significant positive indirect effects of abusive supervision on service sabotage through revenge desire (*B* = 0.22, *LLCI* = 0.12, *ULCI* = 0.34). The same table also indicates the significant positive direct effect of abusive supervision on service sabotage (*B* = 0.47, *LLCI* = 0.67, *ULCI* = 0.54). Besides, it also indicates total effect of abusive supervision on service sabotage (*B* = 0.69, *LLCI* = 0.88, *ULCI* = 0.80). Hence, the table supports our mediation hypothesis.

**TABLE 2:** Results of mediation analysis.

**Antecedents**	**Revenge desire**						**Service sabotage**					
	***B***	***SE***	***t***	**LLCI**	**ULCI**	***R*^2^**	***B***	***SE***	***t***	**LLCI**	**ULCI**	***R*^2^**
						0.34***						0.34***
Constant	1.48	0.34	4.31***	0.80	2.16		0.22	0.37	0.60	–0.51	0.96	
Abusive supervision	0.68	0.09	7.93***	0.51	0.84		0.47	0.10	4.67***	0.27	0.67	
Revenge desire	−	−	−	−	−		0.33	0.07	4.54***	0.19	0.47	
**Control variables**												
Subordinate gender	–0.06	0.10	–0.56	–0.27	0.15		–0.07	0.11	–0.66	–0.29	0.14	
Subordinate age	0.04	0.04	1.09	–0.03	0.12		0.01	0.04	0.06	–0.08	0.08	
Subordinate education	0.01	0.05	0.00	–0.09	0.09		–0.03	0.05	–0.71	–0.13	0.06	
Subordinate experience in service industry	0.01	0.03	–0.19	–0.06	0.05		–0.05	0.03	–1.77	–0.10	0.01	
Customers’ negative events	–0.14	0.04	−3.41***	–0.21	–0.06		0.13	0.04	3.09***	0.05	0.22	

**Total Effect Model**												

**Antecedents**					**Service sabotage**						
					***B***	***SE***		***t***	**LLCI**	**ULCI**		***R*^2^**
												0.27***
Constant					0.71	0.37		1.90	–0.03		1.45	
Abusive supervision					0.69	0.09		7.50***	0.51		0.88	
Revenge desire												
**Control variables**												
Subordinate gender					–0.09	0.11		–0.80	–0.32		0.13	
Subordinate age					0.02	0.04		0.39	–0.07		0.10	
Subordinate education					–0.03	0.05		–0.67	–0.13		0.06	
Subordinate experience in service industry					–0.05	0.03		–1.75	–0.10		0.01	
Customers’ negative events					0.09	0.04		2.00	0.00		0.17	

**Results of direct, indirect, and total, effects of Abusive Supervision on service sabotage.**												

**Predictor**					**Effect**		***SE***		**LLCI**		**ULCI**
**Total effects**								
Abusive supervision on service sabotage					0.69		0.09		0.88		0.80
**Direct effects**								
Abusive supervision on service sabotage					0.47		0.10		0.67		0.54
**Indirect effects**								
Abusive supervision on service sabotage via revenge desire					0.22		0.05		0.12		0.34
**Partially standardized indirect effect**								
Abusive supervision on service sabotage via revenge desire					0.26		0.06		0.15		0.38
**Completely standardized indirect effect**								
Abusive supervision on service sabotage via revenge desire					0.17		0.04		0.09		0.25

N = 63 supervisors and 212 subordinates (Dyadic data); LLCI, Lower level of the 95% confidence interval; ULCI, Upper level of 95% confidence interval; Significant at: ***p < 0.001.

### Test of Moderated Mediation Model

**FIGURE 2:**
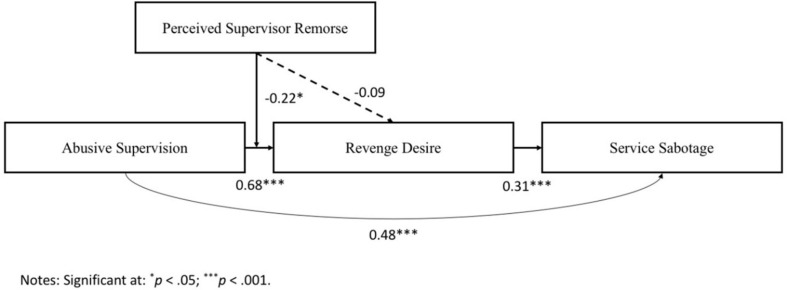
Results of the moderated mediation model.

**FIGURE 3:**
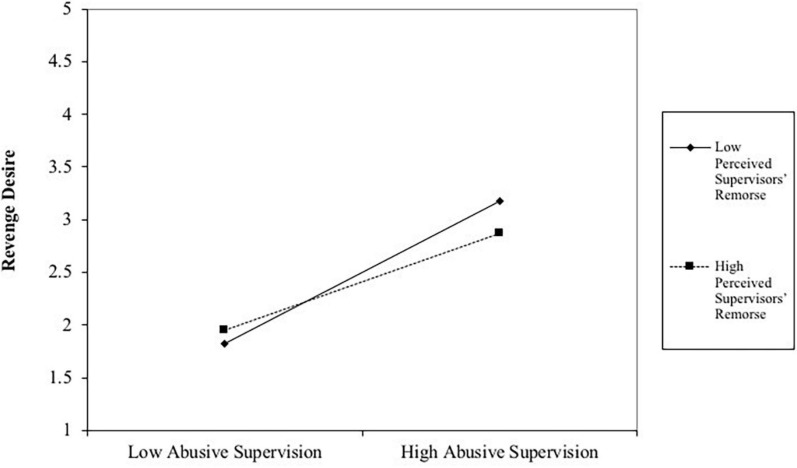
The interaction of abusive supervision and PSR on revenge desire.

Table 3 lists the findings of our moderated mediation model (see also Figure 2). Similar to the result of the simple mediation analyses, we found that abusive supervision is positively correlated with revenge desire (*B* = 0.68, *t* = 8.07, *p* < 0.001) and service sabotage (*B* = 0.48, *t* = 4.85, *p* < 0.001). Revenge desire is also positively correlated with service sabotage (*B* = 0.31, *t* = 4.37, *p* < 0.01). The interaction term of abusive supervision and perceived supervisors’ remorse (PSR) is negative and significant (*B* = −0.22, *t* = −2.28, *p* < 0.05), as indicated in Table 3. Thus, Hypothesis 2a is supported. The positive relationship between abusive supervision and revenge desire is moderated by PSR, such that the positive relationship is weaker when PSR is high. To further support this hypothesis, we plot the interaction term, i.e., Abusive supervision × PSR. Figure 3 is the graphical presentation of the moderating effect of PSR.

**TABLE 3:** Results of the moderated-mediation model analysis.

**Antecedents**	**Revenge desire**						**Service sabotage**					
	***B***	**SE**	***t***	**LLCI**	**ULCI**	***R*^2^**	***B***	**SE**	***t***	**LLCI**	**ULCI**	***R*^2^**
						0.37***						0.35***
Constant	3.86	0.27	14.34***	3.33	4.39		1.90	0.39	4.88***	1.14	2.67	
Abusive supervision	0.68	0.08	8.07***	0.51	0.84		0.48	0.10	4.85***	0.29	0.68	
Revenge desire	–	–	–	–	–		0.31	0.07	4.37***	0.17	0.46	
Perceived supervisors’ remorse	–0.09	0.06	–1.53	–0.21	0.03		–	–	–	–	–	
Abusive supervision X	–0.22	0.10	−2.28*	–0.41	–0.03		–	–	–	–	–	
Perceived supervisors’ remorse												
**Control variables**												
Subordinate gender	–0.08	0.10	–0.81	–0.29	0.12		–0.09	0.11	–0.82	–0.30	0.12	
Subordinate age	0.04	0.04	1.04	–0.04	0.12		0.01	0.04	0.05	–0.08	0.08	
Subordinate education	–0.01	0.04	–0.15	–0.10	0.08		–0.03	0.05	–0.59	–0.12	0.06	
Subordinate experience in service industry	0.01	0.03	0.03	–0.05	0.05		–0.05	0.03	–1.95	–0.10	0.00	
Customers’ negative events	–0.14	0.04	−3.50***	–0.22	–0.06		0.14	0.04	3.32***	0.06	0.22	

N = 63 supervisors and 212 subordinates (Dyadic data); LLCI, Lower level of the 95% confidence interval; ULCI, Upper level of 95% confidence interval; Significant at: *p < 0.05; ***p < 0.001.

To test Hypothesis 2b, we examined the conditional indirect effects of abusive supervision on service sabotage via revenge desire at different values of PSR (−1 SD, M, and +1 SD). Table 4 reveals that the indirect effect of abusive supervision on service sabotage through revenge desire is weak when PSR is high (*B* = 0.16, *LLCI* = 0.06, *ULCI* = 0.27). This effect is strong when PSR is less (B = 0.27, *LLCI* = 0.15, *ULCI* = 0.41). Our moderated mediation (i.e., Hypothesis 2b) is supported. The indirect positive relationship between abusive supervision and service sabotage through revenge desire is moderated by PSR, such that the mediated relationship is weaker when PSR is high.

**TABLE 4:** Results of conditional indirect effects and total conditional effects of abusive supervision on service sabotage at values of perceived supervisors’ remorse.

**Predictor**	**Mediator**	**Moderator**	**Effect**	**SE**	**LLCI**	**ULCI**
Index of the moderated mediation model	Revenge desire		–0.07	0.04	–0.16	–0.01
**Conditional direct effects**						
Abusive supervision on service sabotage	–	Perceived supervisor remorse at −1 SD	0.85	0.11	0.63	1.07
Abusive supervision on service sabotage	–	Perceived supervisor remorse at Mean	0.68	0.08	0.51	0.84
Abusive supervision on service sabotage	–	Perceived supervisor remorse at +1 SD	0.50	0.11	0.28	0.73
**Conditional indirect effects**						
Abusive supervision on service sabotage	Revenge desire	Perceived supervisor remorse at −1 SD	0.27	0.07	0.15	0.41
Abusive supervision on service sabotage	Revenge desire	Perceived supervisor remorse at Mean	0.21	0.05	0.12	0.32
Abusive supervision on service sabotage	Revenge desire	Perceived supervisor remorse at +1 SD	0.16	0.05	0.06	0.27

**N* = 63 supervisors and 212 subordinates (Dyadic data); LLCI, Lower level of the 95% confidence interval; ULCI, Upper level of 95% confidence interval.*

## Discussion

Though numerous studies (Lian et al., 2014; Barnes et al., 2015; Eissa and Lester, 2017; Haggard and Park, 2018) have already been conducted to report annoying outcomes of abusive supervision, there is still some room to further extend this domain through empirical research. One such direction to extend the existing literature is to examine how abusive supervision disturbs service sector organizations by affecting their employees, which results in their service sabotage behavior. This study began with two basic questions, “How abusive supervision results in subordinates’ service sabotage behavior?” and “Do victims’ perception of supervisor remorse weaken the positive association between abusive supervision and subordinates’ service sabotage behavior?” To answer these questions, we borrowed support from social exchange theory and displaced revenge literature. Traditionally, SET provides the major theoretical support to explain a process starting with the social exchange between parties and ending with reciprocal behavioral outcomes. Though several studies have been conducted to examine how the nature of social exchange affects individuals’ behavior, however, how it explains the effect of abusive supervision on subordinates’ behavior with their customers is ignored to date. To fill this gap, this study provides a mechanism to answer our first question, i.e., how abusive supervision results in victims’ exhibition of service sabotage behavior. This study advocates that when employees face abusive supervision, they perceive it as unjust interpersonal mistreatment, which generates revenge desire in them. When victims are filled with revenge desire, they wish to vent out for justice, i.e., the eye for an eye, tooth for a tooth. However, in the case of abusive supervision, there’s an imbalance of power between victim and perpetrator, which makes it difficult for victims to reciprocate abusive behavior. In such a scenario, where victims cannot go for the original perpetrators, they displace their revenge to those whom they perceive to be less powerful. Here, customers are considered an easy target for victims to displace their revenge on. Specifically, this study demonstrates that abused employees do not retaliate against their supervisors; instead, they divert their revenge desire to customers and sabotage services provided to them.

Our findings are in line with prior studies that concluded that victims of interpersonal mistreatment perceive it as they have not been treated suitably and they have no respect in the organization (Cortina and Magley, 2003; Sulea et al., 2012; Hongbo et al., 2019). Such ill thoughts result in destructive behavior on victims’ part followed by negative emotional and psychological episodes. Providing support to prior literature, our findings demonstrate that victims of abusive supervision do not take revenge from their abusive supervisors; rather, they divert their revenge desire toward customers by sabotaging their services. With these arguments, this study lends support to a basic tenant of SET and displaced revenge literature, i.e., such social exchanges that violate the social norms result in undesired behavior, and when recipients cannot strike back at the perpetrator, they divert their reaction toward those who are considered as less harmful.

Besides, this study also demonstrates that abusive supervision does not always result in undesired behavior at victims’ part, which directly answers our second question, i.e., does victims’ perception of supervisor’s remorse weakens the positive impact of abusive supervision on subordinates’ service sabotage behavior? Our results show that detrimental effects of abusive supervision are weaker when supervisors show remorse to their subordinates after being involved in abusive behavior, indicating that PSR plays a crucial role in limiting the adverse effects of abusive supervision on individuals and organizations. Our findings reveal that when the victims perceive their supervisors are remorseful for what they have done, not only by a verbal expression but they also show it by their behavior, restore interactional justice, and help to reduce the victim’s service sabotage behavior by reducing the revenge desire being generated by abusive supervision. Our findings are someway related to Thau et al. (2009) findings, which concluded that the effect of abusive supervision on workplace deviance was stronger when situational uncertainty was high, whereas remorse resolves the uncertainty. Similarly, our stance on the moderating role of remorse in limiting the adversity of abusive supervision is also supported by a prior study (Haggard and Park, 2018) that found that remorse helps to maintain the healthy leader-member exchange by generating a perception of interactional justice.

As children, we were often advised not to hurt others, neither by your words nor by actions, and even if you hurt others, you apologize for that. Alas, most of us forgot these childhood lessons with our growing age. These days, we do not feel any shame while hurting others; we do not even feel the pain that our words may cause to others. There is a need to remind us of those lessons that we were told in our childhood. This study highlights the importance of those lessons and suggests that employees should understand the importance of acknowledging their wrong deeds and apologetic behavior in repairing the loss that their inappropriate behavior causes to others. Efforts to repair the loss by remorse might be a difficult task for managers who perceive themselves as superior and try to link their inappropriate behavior with some external factors. For them, remorse may cause status loss.

In a highly competitive market environment, to gain comparative advantage, all the organizational members should contribute to achieving the organizational goals that are more complex and difficult to achieve without teamwork. In teamwork, members are interdependent, and team performance mainly relies on members’ effort and motivation. So, organizations need to create such a work environment where each individual is considered as an important asset of the organization, which motivates them to contribute to achieving organizational goals. However, in the case of abusive supervision, individuals assume themselves as worthless, which demotivates them. Our study recommends that remorse is one way to restore demotivated individuals’ self-confidence. Organizations should communicate to their supervisors that showing remorse to their subordinates is an expedient strategy to reduce the adversity caused by their interpersonal mistreatment. For that, organizations should develop human-oriented workplace culture; it motivates supervisors to freely express their apology.

## Limitations

We hope that this study highlights a new dimension (perceived supervisor remorse) in abusive supervision literature and motivates future researchers to work on this aspect to enhance its understanding. However, despite the interesting findings, this study has some concerns. First, although this study concludes that perceived supervisor remorse reduces detrimental effects of abusive supervision, however, this effect has some boundary conditions. For instance, the perception of supervisors as trustworthy individuals plays an important role in remorse effectiveness; if a supervisor is repeatedly involved in abusive behavior followed by remorse, then remorse becomes ineffective. In such conditions, subordinates lose their trust in supervisors and attribute their abusive behavior as an intentional behavior rather uncontrollable externality.

Another boundary condition for remorse effectiveness could be the blame attribution. Our findings are based on whether or not employees perceive their supervisors as remorseful but this study did not capture blame attributional perspective, i.e., whether abusive behavior is attributed as intentional behavior or to some unavoidable circumstances. For instance, an experimental study found that forgiveness was less likely awarded when the victims perceive the offense as intentional behavior. Based on this discussion, we suggest future researchers cover this perspective in their research. Third, though this study relies on multi-source data (i.e., supervisors and subordinates), one cannot eliminate the potential issue of common method variance because most of the key variables were reported by the same respondent, e.g., abusive supervision, revenge desire, and PSR. Nevertheless, we tried to mitigate the CMV effect by collecting some information from supervisors, yet our findings might be inflated by CMV. Thus, we encourage future researchers to collect data from multiple sources (i.e., supervisors, subordinates, and customers) to check the robustness of our study’s findings. By doing so, future researchers may collect ratings of service sabotage from supervisors, subordinates, and customers to minimize the CMV concerns as well as to develop a comprehensive understanding of abusive supervision on employees’ service sabotage. Fourth, in our work, we collected data from 21 offices across three provinces, but we didn’t report or examine the influence of potential differences of offices on data analyses and results. We believe that it is necessary to include the potential difference of offices in data analyses to fully understand the relationship between abusive supervision and employee service sabotage. Thus, we encourage future studies to at least consider the response rate for each office and province to check the robustness of our findings.

Fourth, we, from the perspective of interpersonal mistreatment, focused the investigation on the relationship between abusive supervision and victims’ service sabotage behavior via revenge desire at the high vs. low value of perceived supervisors’ remorse. In our study, we followed recent previous studies (e.g., see Tariq et al., 2019) to rely on perceptions and didn’t empirically test the interpersonal mistreatment phenomena. We called for future research to develop a comprehensive model to explore the abusive supervision and victims’ service sabotage behavior by integrating actual interpersonal difference by the supervisors as they impact perceptions by subordinates.

## Conclusion

Social relationships are complex and difficult to maintain, especially between supervisors and subordinates at the workplace. This complexity becomes severe when subordinates experience abusive supervision. This study contributes to the existing literature on abusive supervision and service sabotage by providing a mechanism that explains abusive supervision-service sabotage relationship and also examines the effect of PSR on victims’ behavior with their customers. This study concludes that when subordinates see their supervisors are remorseful for their hurtful behavior it mitigates the negative effects (service sabotage) of the abusive behavior by weakening the victims’ revenge desire. We sincerely hope that other researchers will join us in studying remorse at the workplace.

## Data Availability Statement

The datasets generated for this study are available on request to the corresponding author.

## Ethics Statement

The studies involving human participants were reviewed and approved by the Ethics Committee at Jiangsu University. Participants’ written informed consent to participate in this study was inferred through the completion of the survey.

## Author Contributions

All authors equally contributed to conception and design, acquisition of data, analysis and interpretation of data. They are also drafted the article for important intellectual content. All authors approved final version to be published and agreement to be accountable for all aspects of the work in ensuring that questions related to the accuracy or integrity of any part of the work are appropriately investigated and resolved.

## Conflict of Interest

The authors declare that the research was conducted in the absence of any commercial or financial relationships that could be construed as a potential conflict of interest.
