# Corrigendum: I Will Hurt You for This, When and How Subordinates Take Revenge From Abusive Supervisors: A Perspective of Displaced Revenge

**DOI:** 10.3389/fpsyg.2021.672074

**Published:** 2021-05-26

**Authors:** Li Hongbo, Muhammad Waqas, Hussain Tariq, Atuahene Antwiwaa Nana Abena, Opoku Charles Akwasi, Sheikh Farhan Ashraf

**Affiliations:** ^1^School of Management, Jiangsu University, Zhenjiang, China; ^2^NUST Business School, National University of Sciences and Technology, Islamabad, Pakistan; ^3^Management Science and Engineering, School of Management, Jiangsu University, Zhenjiang, China

**Keywords:** abusive supervision, social exchange theory, displaced revenge, service sabotage, perceived supervisors' remorse

In the original article, there were mistakes in Figures 2 and 3 as published. For Figure 2, the values mentioned in the figure were wrong. For Figure 3, abbreviations were used, and the data were wrongly presented. The corrected figures appear below.

**Figure 2:**
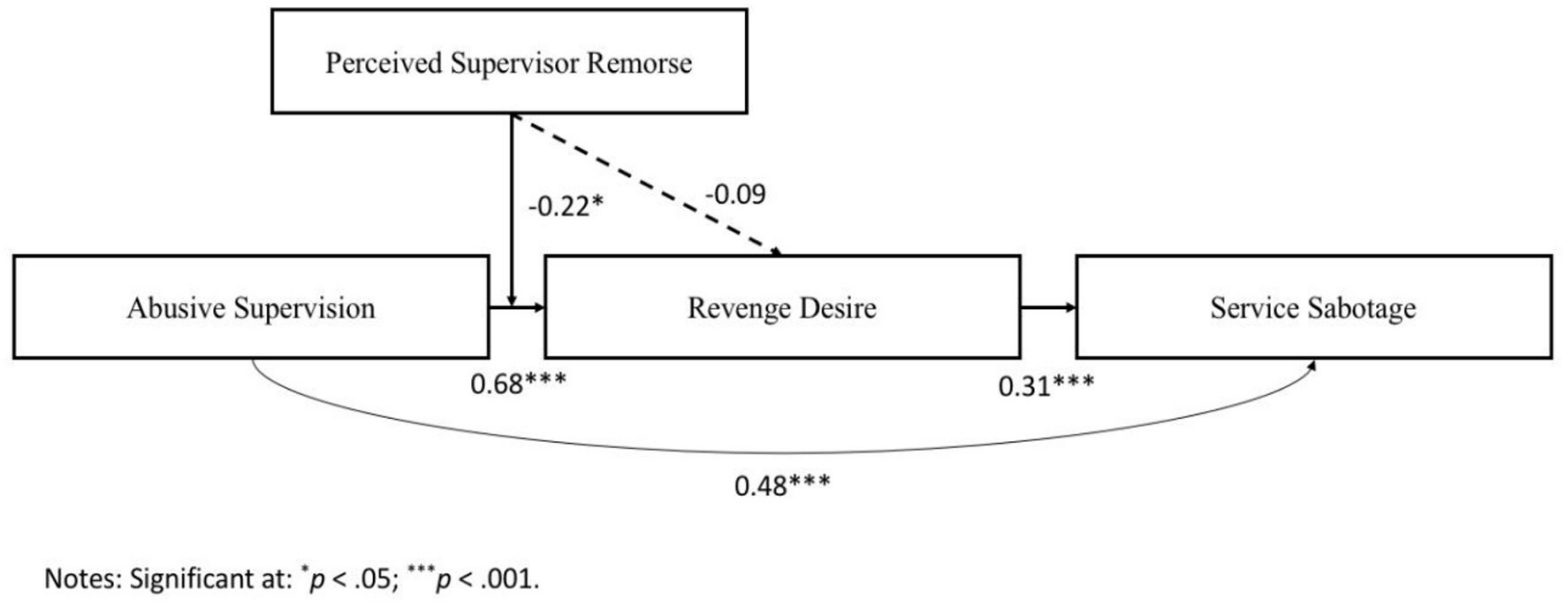
Results of the moderated mediation model.

**Figure 3:**
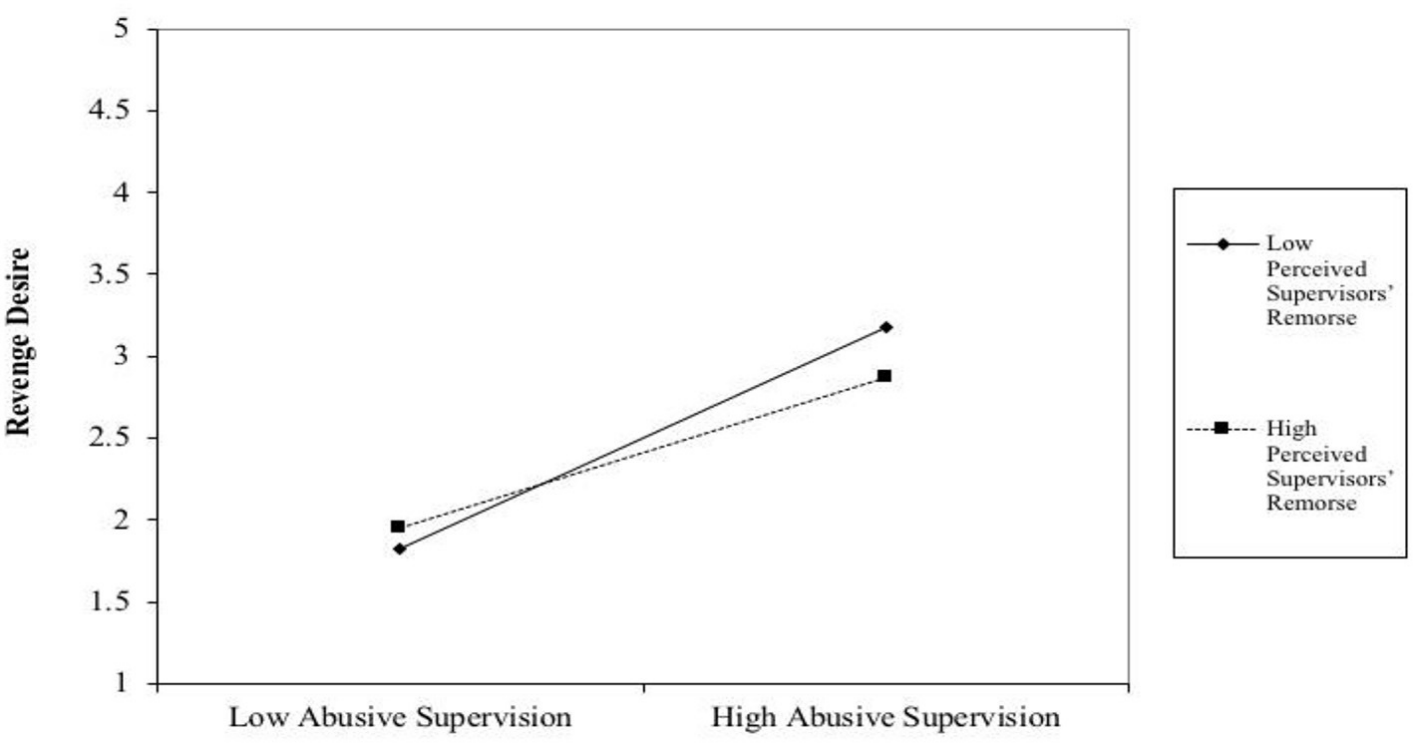
The interaction of abusive supervision and PSR on revenge desire.

Additionally, in the original article, there were mistakes in Tables 1, 2, 3, and 4 as published. The values reported in the tables were wrong and some relevant information was missing. The corrected tables appear below.

**Table 1:** Intercorrelations, descriptive statistics, and estimated reliabilities among the latent variables.

**Variables**	**M**	**SD**	**1**	**2**	**3**	**4**	**5**	**6**	**7**	**8**	**9**
Subordinate gender^a^	1.32	0.46	–								
Subordinate age^b^	3.06	1.21	−0.10	–							
Subordinate education^c^	3.08	1.21	−0.03	0.11	–						
Subordinate experience in service industry^d^	2.22	1.89	−0.23^**^	−0.06	−0.03	–					
Customers' negative events	2.13	1.20	−0.01	−0.04	−0.11	0.06	(0.70)				
Abusive supervision	3.43	0.64	−0.02	0.13	0.49^**^	−0.05	−0.09	(0.88)			
Revenge desire	3.55	0.83	−0.04	0.14^*^	0.28^**^	−0.04	−0.24^**^	0.54^**^	(0.76)		
Service sabotage	3.00	0.86	−0.04	0.08	0.20^**^	−0.11	0.07	0.49^**^	0.46^**^	(0.77)	
Perceived supervisor remorse	3.37	0.78	−0.02	−0.06	−0.09	0.03	−0.08	−0.01	−0.08	0.01	(0.74)

*N = 63 supervisors and 212 subordinates (Dyadic data); Significant at:*

* *p < 0.05;*

** *p < 0.01; figures in parentheses are alpha internal consistency reliabilities*.

a *Subordinate gender: 1 = Male, 2 = Female;*

b *Subordinate age: 1 = Less than 24 years, 2 = 26–30 years, 3 = 31–35 years, 4 = 36–40 years, 5 = more than 40 years;*

c *Subordinate education: 1 = Primary education, 2 = High school, 3 = College education, 4 = Vocational education 5 = Others;*

d *Subordinate working experience in service industry = 1 = Less than 1 year, 2 = 1–3 years, 3 = 4–6 years, 4 = 7–9 years, more than 9 years*.

**Table 2:** Results of mediation analysis.

**Antecedents**	**Revenge desire**						**Service sabotage**					
	***B***	***SE***	***t***	**LLCI**	**ULCI**	***R^**2**^***	***B***	***SE***	***t***	**LLCI**	**ULCI**	***R^**2**^***
						0.34^***^						0.34^***^
Constant	1.48	0.34	4.31^***^	0.80	2.16		0.22	0.37	0.60	−0.51	0.96	
Abusive supervision	0.68	0.09	7.93^***^	0.51	0.84		0.47	0.10	4.67^***^	0.27	0.67	
Revenge desire	–	–	–	–	–		0.33	0.07	4.54^***^	0.19	0.47	
**Control variables**												
Subordinate gender	−0.06	0.10	−0.56	−0.27	0.15		−0.07	0.11	−0.66	−0.29	0.14	
Subordinate age	0.04	0.04	1.09	−0.03	0.12		0.01	0.04	0.06	−0.08	0.08	
Subordinate education	0.01	0.05	0.00	−0.09	0.09		−0.03	0.05	−0.71	−0.13	0.06	
Subordinate experience in service industry	0.01	0.03	−0.19	−0.06	0.05		−0.05	0.03	−1.77	−0.10	0.01	
Customers' negative events	−0.14	0.04	−3.41^***^	−0.21	−0.06		0.13	0.04	3.09^***^	0.05	0.22	
**Total Effect Model**												
**Antecedents**				**Service sabotage**								
				***B***	***SE***		***t***		**LLCI**	**ULCI**		***R**^**2**^*
												0.27^***^
Constant				0.71	0.37		1.90		−0.03		1.45	
Abusive supervision				0.69	0.09		7.50^***^		0.51		0.88	
Revenge desire												
**Control variables**												
Subordinate gender				−0.09	0.11		−0.80		−0.32		0.13	
Subordinate age				0.02	0.04		0.39		−0.07		0.10	
Subordinate education				−0.03	0.05		−0.67		−0.13		0.06	
Subordinate experience in service industry				−0.05	0.03		−1.75		−0.10		0.01	
Customers' negative events				0.09	0.04		2.00		0.00		0.17	

**Table:** 

**Results of direct, indirect, and total, effects of Abusive Supervision on service sabotage**.												
**Predictor**	**Effect**	***SE***	**LLCI**	**ULCI**
**Total effects**				
Abusive supervision on service sabotage	0.69	0.09	0.88	0.80
**Direct effects**				
Abusive supervision on service sabotage	0.47	0.10	0.67	0.54
**Indirect effects**				
Abusive supervision on service sabotage via revenge desire	0.22	0.05	0.12	0.34
**Partially standardized indirect effect**				
Abusive supervision on service sabotage via revenge desire	0.26	0.06	0.15	0.38
**Completely standardized indirect effect**				
Abusive supervision on service sabotage via revenge desire	0.17	0.04	0.09	0.25

*N* = 63 supervisors and 212 subordinates (Dyadic data); LLCI, Lower level of the 95% confidence interval; ULCI, Upper level of 95% confidence interval; Significant at:

*** *p < 0.001*.

**Table 3:** Results of the moderated-mediation model analysis.

**Antecedents**	**Revenge desire**						**Service sabotage**					
	***B***	**SE**	***t***	**LLCI**	**ULCI**	***R^**2**^***	***B***	**SE**	***t***	**LLCI**	**ULCI**	***R^**2**^***
						0.37^***^						0.35^***^
Constant	3.86	0.27	14.34^***^	3.33	4.39		1.90	0.39	4.88^***^	1.14	2.67	
Abusive supervision	0.68	0.08	8.07^***^	0.51	0.84		0.48	0.10	4.85^***^	0.29	0.68	
Revenge desire	–	–	–	–	–		0.31	0.07	4.37^***^	0.17	0.46	
Perceived supervisors' remorse	−0.09	0.06	−1.53	−0.21	0.03		–	–	–	–	–	
Abusive supervision X Perceived supervisors' remorse	−0.22	0.10	−2.28^*^	−0.41	−0.03		–	–	–	–	–	
**Control variables**												
Subordinate gender	−0.08	0.10	−0.81	−0.29	0.12		−0.09	0.11	−0.82	−0.30	0.12	
Subordinate age	0.04	0.04	1.04	−0.04	0.12		0.01	0.04	0.05	−0.08	0.08	
Subordinate education	−0.01	0.04	−0.15	−0.10	0.08		−0.03	0.05	−0.59	−0.12	0.06	
Subordinate experience in service industry	0.01	0.03	0.03	−0.05	0.05		−0.05	0.03	−1.95	−0.10	0.00	
Customers' negative events	−0.14	0.04	−3.50^***^	−0.22	−0.06		0.14	0.04	3.32^***^	0.06	0.22	

*N = 63 supervisors and 212 subordinates (Dyadic data); LLCI, Lower level of the 95% confidence interval; ULCI, Upper level of 95% confidence interval; Significant at:*

* *p < 0.05;*

*** *p < 0.001*.

**Table 4:** Results of conditional indirect effects and total conditional effects of abusive supervision on service sabotage at values of perceived supervisors' remorse.

**Predictor**	**Mediator**	**Moderator**	**Effect**	**SE**	**LLCI**	**ULCI**
Index of the moderated mediation model	Revenge desire		−0.07	0.04	−0.16	−0.01
**Conditional direct effects**						
Abusive supervision on service sabotage	–	Perceived supervisor remorse at −1 SD	0.85	0.11	0.63	1.07
Abusive supervision on service sabotage	–	Perceived supervisor remorse at Mean	0.68	0.08	0.51	0.84
Abusive supervision on service sabotage	–	Perceived supervisor remorse at +1 SD	0.50	0.11	0.28	0.73
**Conditional indirect effects**						
Abusive supervision on service sabotage	Revenge desire	Perceived supervisor remorse at −1 SD	0.27	0.07	0.15	0.41
Abusive supervision on service sabotage	Revenge desire	Perceived supervisor remorse at Mean	0.21	0.05	0.12	0.32
Abusive supervision on service sabotage	Revenge desire	Perceived supervisor remorse at +1 SD	0.16	0.05	0.06	0.27

*N = 63 supervisors and 212 subordinates (Dyadic data); LLCI, Lower level of the 95% confidence interval; ULCI, Upper level of 95% confidence interval*.

Lastly, in the original article, there were several errors in the text.

In *Measures, Abusive Supervision, Paragraph 1*, alpha reliability was wrongly reported. The corrected paragraph appears below.

**Abusive Supervision**

Abusive supervision was measured using Tepper (2000) 15-item scale, which asks respondents to indicate how often their supervisors used certain behaviors (1 = never, 5 = always). Sample items include, “My supervisor tells me my thoughts or feelings are stupid,” and “Is rude to me.” Alpha reliability was 0.88.

In *Measures, Employee Service Sabotage, Paragraph 1*, the Cronbach's α was wrongly reported. The corrected paragraph appears below.

**Employee Service Sabotage**

Many service sabotage measures were created to use in a call center setting. While, there is big dissimilarity in the services offered by call center agents, and those who deal face-to-face with the customers. Along with such doubts in mind, we calculated service sabotage using the Chi et al. (2013) three-item scale. Statements such as “mistreating customers deliberately” were measured on a five-point attribution scale (1 = never, and 5 = always). The Cronbach's α for this scale was 0.77.

In *Measures, Perceived Supervisor Remorse, Paragraph 1*, alpha reliability was wrongly reported. The corrected paragraph appears below.

**Perceived Supervisor Remorse**

Perception of supervisor remorse was measured using Haggard and Park (2018) 10-item scale, which asks respondents to indicate how often their supervisors used certain behaviors after they (supervisors) had done something hurtful: “Admitted that his/her behavior was unacceptable,” “Took responsibility for his/her hurtful behavior,” “Asked what he/she could do to repair the damage to your relationship,” and “Expressed that he/she felt bad about how his/her behavior affected you” (1 = never, and 5 = always). Alpha reliability was 0.74.

In *Results, Descriptive Statistics, Paragraph 1*, the *r* values were wrongly reported. The corrected paragraph appears below.

**Descriptive statistics**

Table 1 provides our study's descriptive statistics (standard deviations, means, and estimated coefficient alpha values) and intercorrelations. The preliminary analyses support our hypotheses i.e., abusive supervision is positively related to revenge desire (*r* = 0.54, *p* < 0.01) and service sabotage (*r* = 0.49, *p* < 0.01). Revenge desire is also positively related to service sabotage (*r* = 0.46, *p* < 0.01).

In *Results, Test of Mediation, Paragraph 1*, several values were wrongly reported. The corrected paragraph appears below.

**Test of mediation**

Table 2 presents the findings of the mediation test. Abusive supervision is positively correlated with revenge desire (*B* = 0.68, *t* = 7.93, *p* < 0.001) and service sabotage (*B* = 0.47, *t* = 4.67, *p* < 0.001). Revenge desire is also positively correlated with service sabotage (*B* = 0.33, *t* = 4.54, *p* < 0.001). Table 2 also indicates the significant positive indirect effects of abusive supervision on service sabotage through revenge desire (*B* = 0.22, LLCI = 0.12, ULCI = 0.34). The same table also indicates the significant positive direct effect of abusive supervision on service sabotage (*B* = 0.47, LLCI = 0.67, ULCI = 0.54). Besides, it also indicates total effect of abusive supervision on service sabotage (*B* = 0.69, LLCI = 0.88, ULCI = 0.80). Hence, the table supports our mediation hypothesis.

In *Results, Test of the Moderated Mediation Model, Paragraphs 1 and 2*, several values were wrongly reported. The corrected paragraphs appear below.

**Test of moderated mediation model**

Table 3 lists the findings of our moderated mediation model (see also Figure 2). Similar to the result of the simple mediation analyses, we found that abusive supervision is positively correlated with revenge desire (*B* = 0.68, *t* = 8.07, *p* < 0.001) and service sabotage (*B* = 0.48, *t* = 4.85, *p* < 0.001). Revenge desire is also positively correlated with service sabotage (*B* = 0.31, *t* = 4.37, *p* < 0.01). The interaction term of abusive supervision and perceived supervisors' remorse (PSR) is negative and significant (*B* = −0.22, *t* = −2.28, *p* < 0.05), as indicated in Table 3. Thus, Hypothesis 2a is supported. The positive relationship between abusive supervision and revenge desire is moderated by PSR, such that the positive relationship is weaker when PSR is high. To further support this hypothesis, we plot the interaction term, i.e., Abusive supervision × PSR. Figure 3 is the graphical presentation of the moderating effect of PSR.

To test Hypothesis 2b, we examined the conditional indirect effects of abusive supervision on service sabotage via revenge desire at different values of PSR (−1 SD, M, and +1 SD). Table 4 reveals that the indirect effect of abusive supervision on service sabotage through revenge desire is weak when PSR is high (*B* = 0.16, LLCI = 0.06, ULCI = 0.27). This effect is strong when PSR is less (*B* = 0.27, LLCI = 0.15, ULCI = 0.41). Our moderated mediation (i.e., Hypothesis 2b) is supported. The indirect positive relationship between abusive supervision and service sabotage through revenge desire is moderated by PSR, such that the mediated relationship is weaker when PSR is high.

The authors apologize for these errors and state that they do not change the scientific conclusions of the article in any way. The original article has been updated.
